# Psychiatric and neurodevelopmental diagnoses in adolescence and adulthood over-indebtedness among Finns born in 1987

**DOI:** 10.1093/eurpub/ckac126

**Published:** 2022-10-10

**Authors:** Aapo Hiilamo, Markus Keski-Säntti, Sami Pirkola, Tea Lallukka, Antti Kääriälä

**Affiliations:** Faculty of Social Sciences, University of Helsinki, Helsinki, Finland; Itla Children’s Foundation, Helsinki, Finland; Finnish Institute for Health and Welfare (THL), Helsinki, Finland; Finnish Institute for Health and Welfare (THL), Helsinki, Finland; Faculty of Social Sciences, Tampere University and Pirkanmaa Hospital District, Department of Psychiatry, Tampere, Finland; Department of Public Health, Faculty of Medicine, University of Helsinki, Helsinki, Finland; Finnish Institute for Health and Welfare (THL), Helsinki, Finland; Research Centre for Child Psychiatry and INVEST Research Flagship, University of Turku, Turku, Finland

## Abstract

**Background:**

Adolescence psychiatric and neurodevelopmental diagnoses are common but their link to adulthood over-indebtedness is unknown. This study aims to determine this relationship and explores the possible mediating role of upper secondary education completion.

**Methods:**

We analyzed the 1987 Finnish Birth Cohort, which consisted of a complete census of children born in Finland in 1987 and registered in the Medical Birth Register (*n* = 53 743). Records of debt payment defaults, at the age of 33, were used as a measure of over-indebtedness. Adolescent psychiatric and neurodevelopmental diagnoses at ages 13–17 were derived from the national hospital discharge register. Inverse probability treatment weighting was used to investigate the role of pre-exposure variables in this relationship, and the mediating role of upper secondary education completion.

**Results:**

Compared to unexposed individuals, those affected by an adolescent psychiatric or neurodevelopmental diagnosis had a 15 percentage points higher prevalence of over-indebtedness in adulthood. This association was more common for males and was additionally notably strong for suicidality and conduct and oppositional disorders. Controlling for measured potential confounding factors, the diagnoses were linked to a 11-percentage point (95% confidence interval 9–12) higher risk of over-indebtedness. Completing at least upper secondary education reduced this effect by some 39%.

**Conclusion:**

People with psychiatric and neurodevelopmental disorders diagnosed in adolescence are at elevated risk of over-indebtedness in adulthood. Recognizing this high risk may help in efforts to prevent further debt problems. Better education may serve as a protective factor against over-indebtedness and perhaps similar other behavioural consequences.

## Introduction

A substantial proportion of children and adolescents experience psychiatric and neurodevelopmental conditions.^1^ Some 11% of 5- to 14-year-olds and 15% of 15- to 19-year-olds of experience a mental disorder.^2^ In Finland, almost one-fifth of a recent full cohort had experienced mental or neurodevelopmental diagnoses before their 18th birthday.^3^^,^^4^ The most frequent disorders among people aged less than 18 are anxiety disorders, behavioural disorders, mood disorders and substance use-related disorders.^5^

The long-term socioeconomic consequences of these disorders are important to understand in order to assess the benefits of preventing the disorders early on. It is well established that adolescent psychiatric diagnoses associate with a broad range of negative adulthood socioeconomic outcomes, including for example, lower income,^6^ unemployment^7^ and a shorter education trajectory.^8^ However, the research has ignored over-indebtedness as a potential outcome of these diagnoses. Over-indebtedness is a marker of severe financial distress,^9^ causes social and economic exclusion and may further exacerbate mental health problems.^10^ Over-indebtedness, e.g. with records of unpaid debts, prevents access to good credit, housing, Internet and phone subscriptions, and, in some circumstances, employment.^11^ Over-indebtedness thus incurs substantial costs for both individuals themselves and society.

In addition to the prevention, it is also important to consider potential interventions for people already affected by mental and neurodevelopmental disorders to help mitigate the adverse later-life consequences. Adolescent psychiatric disorders reduce the probability of completing upper secondary education,^12–16^ which is an intermediate outcome that further elevates the risk of unfavourable socioeconomic outcomes. In this study, we thus suspect that education may serve as a key factor through which the link between mental disorders in adolescence and over-indebtedness could be mitigated.

Using data from the full 1987 population birth cohort of Finland, we estimate the effect of adolescent psychiatric diagnoses (in Supplementary figure S1, exposure) on over-indebtedness (outcome) while adjusting for a range of pre-treatment factors (confounding). We also aim to quantify the extent to which this effect could be eliminated by an intervention under which all people would complete at least upper secondary education.

## Methods

### Data

This study analyses the Finnish birth cohort data of 1987 (FBC 1987).^17^ FBC 1987 consists of all people born in Finland and who survived the first week of their lives. FBC 1987 was constructed by combining administrative registers for the cohort members and their biological parents. The unique Finnish personal identification number assigned to each resident allows for reliable matching of different administrative records.^18^ Register data provide a study design with a low rate of attrition and missing data. For this study, we used variables derived from several registers that are listed in the Supplementary materials. All the used registers have full coverage of the cohort members who have not emigrated. FBC 1987 has received approval from the ethical committee of the Finnish Institute for Health and Welfare. Permission was obtained from the data holders to use the register data for scientific purposes. More details are available elsewhere.^17^

The original birth cohort consisted of 59 476 people, from which we excluded the deceased, those who had emigrated, people with intellectual disabilities (ICD-10 and ICD-9 codes starting F0, F7, 317, 318 and 319), people with records of conservatorship, thus not at risk of over-indebtedness, and people with missing confounding variables. After these exclusions, our final sample consisted of 53 743 people (Supplementary table S2).

### Outcome: over-indebtedness

We used debt payment default entry records as an administrative marker of over-indebtedness.^9^ The debt payment entry record data were obtained from a nationwide private credit register holder in Finland. The country has a national negative credit registry, that is, all debt payment defaults are registered (instead of more specific details of credit score). Debt payment default entry records are the main data source for creditworthiness checks concerning credit, rental and labour markets. Debt payment default entry records may be caused by the following: court decisions on unpaid private debts, unpaid debts to the state or quasi-state organizations (unpaid priority debts), long periods of debts in distraints, debt settlement procedures or, in some circumstances, an unpaid credit debt over 60 days late. In any case, debt payment records are a result of oversights of multiple payment reminders, usually over period of several months, and are rarely caused by a distraction. Thus, these records are a sign of over-indebtedness.^9^

Our outcome variable is dichotomous, assigning a value of one for people with at least one debt payment entry record, or zero otherwise. Most people with debt payment entry records have multiple records simultaneously. However, we decided to use a dichotomous measure given that the negative effects of credit records already stem from the first record. The debt payment default entry records were measured on 1 December 2020.

### Exposure: adolescent psychiatric and neurodevelopmental diagnosis

Psychiatric and neurodevelopmental diagnoses were measured in 2000–04 when the cohort members were 13–17 years old, on average. Data on the diagnoses were derived from the Care Register for Health Care. This register source consisted of all specialized inpatient and outpatient hospital visits in public hospitals. These records have been used widely in earlier research.^4^ A dichotomous variable was computed, taking the value one when a person had at least one record with a mandatory primary or secondary diagnosis of a psychiatric or neurodevelopmental disorder, and zero otherwise. The psychiatric and neurodevelopmental diagnoses included all F codes, suicidality and self-harm (X60–X84, Z72.8, Z91.5 and Y87.0).

We also investigated specific adolescent diagnoses. Using ICD-10 codes, we created the following groups: conduct and oppositional disorders (F90.1, F91, F92), depression and anxiety (F32–F34, F38–F41 (excluding F41.2), F42, F93, F94), eating disorders (F50), neurodevelopmental conditions (F80–84, F90, F95), psychotic and bipolar disorders (F20–25, F28–31), self-harm and suicidality (X60–X84, Z72.8, Z91.5, Y87.0), substance-related disorders (F10, F11–19) and other F-diagnoses.

### Mediator: completion of secondary education outcomes

In Finland, until 2020, the compulsory education age has been 16. Education is free of charge. Enrolment in upper secondary education happens around the age of 16 and was voluntary until 2020. Upper secondary education can take the form of either general upper secondary or vocational education and training. More info on the education system is available elsewhere. e.g^19^ We measured whether a person had completed secondary education. A dichotomous variable took a value of one when a person had records of educational qualifications by 2015 (when the cohort members were 27 years old on average) and zero otherwise.

### Confounders

All variables capturing confounding were measured in 1987–99, before the exposure variable was measured. The birth sex was coded for men and women. Childhood characteristics included age (continuous), psychiatric diagnoses in 1987–93, i.e. at ages 0–6 (dichotomous variable), psychiatric diagnoses in 1994–99, i.e. at ages 7–12 (dichotomous), any hospital visit due to somatic diagnoses in 1987–93 (dichotomous), any hospital visit due to somatic diagnoses in 1994–99 (dichotomous), the death of a parent in 1987–99 (dichotomous), placement outside home before the age of 13 (dichotomous), birth weight (continuous) and the parents’ use of social assistance in 1987–99 (continuous). Parental characteristics separately for mothers and fathers included their age at birth (continuous), highest educational qualifications (categorical with four categories), any hospital visit due to somatic diagnoses in 1987–99 (dichotomous, pregnancy-related diagnosis were not included), psychiatric diagnoses in 1987–99 (dichotomous), the number of hospital visits in 1987–99 excluding pregnancy-related visits (continuous), work disability in 1987–99 (dichotomous), unemployment duration in 1987–99 (continuous). Additionally, we included a dichotomous variable on whether there was no data on a person’s father. For technical reasons, fathers’ variables were coded to zero when there was no data on the person’s father. The low number of people without any data on their mothers was not included in the sample due to missing confounder information. We also included municipal/city-level unemployment rate and categorical variable of the hospital district (we merged all hospital districts with fewer than 1000 persons into one category). We provide the register sources of these variables in Supplementary table S1.

### Statistical analyses

We first calculated the descriptive statistics by psychiatric and neurodevelopmental diagnosis and the unadjusted population association between the variables.

We then estimated the extent to which this association remained unexplained by potential confounding factors and explored the mediating role of upper secondary education completion in accordance our causal model shown in Supplementary figure S1. We first present our parameters of interest. $Y_{i}$ denotes the over-indebtedness, treatment, ($Y_{i}=0\;\mathrm{or}\;1$ for no, yes, respectively), which is observed for all persons of the population of interest. Each person *i* is also considered by whether they have had an adolescent psychiatric or neurodevelopmental diagnose ($D_{i}=0\;\mathrm{or}\;1$ for no and yes, respectively) and by an intermediate variable of upper secondary education completion, $M_{i}$ ($M_{i}=0\;\mathrm{or}\;1$ for not completing and completing secondary education, respectively). Y(dm) denotes the potential outcome when exposure, here an adolescent psychiatric diagnose, D is, possibly contrary to observed, set to d and M, possibly contrary to observed, set to m. Let M(d) be the potential value of the mediator when, possibly contrary to observed, D is set d. E[Y(dm)] is the average of the potential outcome in the population.

Our aim was to estimate the total effect (TE), controlled direct effects (CDEs) and proportion eliminated (PE). TE compares the means of the potential outcomes when D is set to d but M is not manipulated: TE = E[Y(D = 1), M(D = 1)] − E[Y(D = 0), M(D = 0)]. CDE compares the population means of the potential outcomes when D is manipulated and M is fixed to a value of 1 for all: CDI = E[Y(D = 1, M = 1] − E[Y(0, M = 1)]. CDE thus measures the effect of adolescent psychiatric or neurodevelopmental diagnoses on over-indebtedness in a scenario under which all have completed at least upper secondary education (i.e. universal upper secondary education completion). Finally, PE = [TE − CDE(M = 1)]/TE illustrates the proportion of the effect, on the additive scale, that this scenario could possibly eliminate.^20^

These three parameters of interest were estimated using inverse probability treatment weighting (IPW) techniques. IPW aims to break the link between the treatment and observed confounding variables by balancing the distribution of observed pre-treatment characteristics between the compared groups. The IPW technique, for causal interpretation, requires assumptions of no unmeasured confounding assumptions and no misspecification of the treatment model.^21^

We fitted the treatment model, that is, a logistic model regressing adolescent psychiatric and neurodevelopmental diagnoses on all measured confounding variables. We included continuous variables with several splines to allow non-linear predictions. The fitted probability from this model was used as an estimate of the probability of treatment. Then each person was weighted by the inverse of the probability treatment that they received, i.e. 1/(the probability of treatment) for the treated and 1/(1 − the probability of treatment) for the comparison group. The means of potential outcomes and TE were estimated using weighted means and their difference from the sample.

In estimating CDE, we used the approach described by Vanderweele.^22^ Additional weights were calculated for the inverse probability of the mediator. In a logistic regression model predicting the completion of upper secondary education, we used the same variables as in the treatment model and additionally included the inverse probability treatment weights as a continuous predictor and the treatment variable as a dichotomous predictor. The final weights for the controlled direct effects were constructed by multiplying the inverse of the probability of treatment weights and the inverse of the probability of mediator weights. CDEs were then obtained from a weighted logistic regression in which the over-indebtedness outcome was regressed for the treatment, mediator and their interaction. Finally, the proportion eliminated was calculated by comparing the total effects and controlled direct effects. 95% confidence intervals were calculated using bootstrapping with 500 replications. In the Supplementary materials, we report the balance of characteristics as recommended.^23^ The Stata 16 software program was used.^24^

## Results

Table 1 shows the characteristics of the cohort. Some 6% of the cohort had had an adolescent psychiatric or neurodevelopmental diagnosis with a higher prevalence among women. Some 91% of the cohort had completed upper secondary education with a lower share among men. The prevalence of over-indebtedness in adulthood was 11% (table 2). For the group without an adolescent diagnosis, the figure was 10% and for the group with an adolescent diagnosis, the proportion was 2.51-fold at 26%.

**Table 1:** Description of the sample by sex. The Finnish Birth Cohort 1987 (*n* = 53 743)

	All	Women	Men
Exposure: Psychiatric diagnosis at ages 13–17 (in 2000–4) (%)	6	8	5
Mediator: Upper secondary education qualifications (%)	91	93	88
Control variables			
Age when over-indebtedness was measured, mean	32.51	32.51	32.51
Psychiatric diagnosis at ages 0–6 (in 1987–93) (%)	1	>1	1
Psychiatric diagnosis at ages 7–12 (in 1994–9) (%)	1	>1	1
Any somatic hospital visit at ages 0–6 (in 1987–93) (%)	38	33	42
Any somatic hospital visit at ages 7–12 (in 1994–9) (%)	35	32	38
Mother's age at birth (mean)	28.92	28.90	28.93
Mother's any somatic hospital visit at ages 0–12 of the cohort member (in 1987–99) (%)	70	69	70
Mother's psychiatric diagnosis at ages 0–12 of the cohort member (in 1987–99) (%)	4	4	4
Mother's hospitalization visits at ages 0–12 of the cohort member (in 1987–99) (mean)	3.61	3.56	3.66
Mother's work disability at ages 0–12 of the cohort member (in 1987–99) (%)	1	1	1
Mother's unemployment duration at ages 0–12 of the cohort member (in 1987–99) (mean)	0.57	0.57	0.57
Father’s age at birth (mean)	30.91	30.88	30.95
Father's somatic diagnosis at ages 0–12 of the cohort member (in 1987–99) (%)	57	58	57
Father's psychiatric diagnosis at ages 0–12 of the cohort member (in 1987–99) (%)	5	5	5
Father’s hospitalization visits at ages 0–12 of the cohort member (in 1987–99) (mean)	2.94	2.96	2.93
Father's work disability at ages 0–12 of the cohort member (in 1987–99) (%)	2	2	2
Father's unemployment duration at ages 0–12 of the cohort member (in 1987–99), mean	0.52	0.52	0.51
Death of a parent at ages 0–12 of the cohort member (in 1987–99) (%)	3	3	2
Parental use of social assistance at ages 0–12 of the cohort member (in 1987–99) mean months	9.21	9.42	9.02
Birth sex, women (%)	51	0	1.00
No data on the father's age (%)	1	1	1
Placement outside home before the age of 13 (%)	1	1	1
Local unemployment rate in 1999			
Unemployment rate (%)	14.51	14.54	14.47
Birth weight (g)			
Mean (g)	3573	3506	3637

After taking into account the confounding variables, the estimated total effect of any psychiatric and neurodevelopmental diagnosis on over-indebtedness was a 11 percentage points on the risk difference (RD) scale (95% confidence interval 9–12) and 2.01 on the risk ratio (RR) scale (1.86–2.17, table 3). Controlled direct effects suggested that this association operated to a significant extent due to a lower rate of secondary education completion. An intervention under which all members of the population would complete at least upper secondary education would reduce the estimated effect by some 39% (95% CI = 28–51%) to 6 percentage points on the RD scale.

**Table 2:** Prevalence of over-indebtedness by sex and adolescence psychiatric diagnosis The Finnish Birth Cohort 1987 (*n* = 53 743)

	All	Men	Women
Prevalence of over-indebtedness—all	0.11	0.13	0.09
People without an adolescent psychiatric diagnosis	0.10	0.12	0.08
People with an adolescent psychiatric diagnosis	0.26	0.32	0.21
Risk difference	0.15	0.20	0.13
Risk ratio	2.51	2.71	2.52
Additive moderation			−0.07
Multiplicative moderation			0.93

**Table 3:** Estimates means of potential outcomes (PO), their differences estimated via inverse probability treatment weighting analyses. The Finnish Birth Cohort 1987 (*n* = 53 743)

	All	Men	Women
Total effects			
A: PO mean psychiatric diagnosis	0.21	0.25	0.17
B: PO mean no psychiatric diagnosis	0.10	0.12	0.09
Total causal effect: Risk difference (A–B)	0.11	0.13	0.08
95% confidence interval	0.09–0.12	0.10–0.16	0.06–0.10
Risk ratio (A/B)	2.01	2.06	1.93
95% confidence interval	1.86–2.17	1.83–2.30	1.70–2.15
Controlled direct effect when all complete upper secondary education			
Controlled risk difference	0.06	0.07	0.06
95% confidence interval	0.05–0.08	0.04–0.10	0.04–0.08
Proportion eliminated	0.39	0.46	0.27
95% confidence interval	0.28–0.51	0.30–0.62	0.14–0.41
*N*	53 743	27 601	26 142

**Figure 1:**
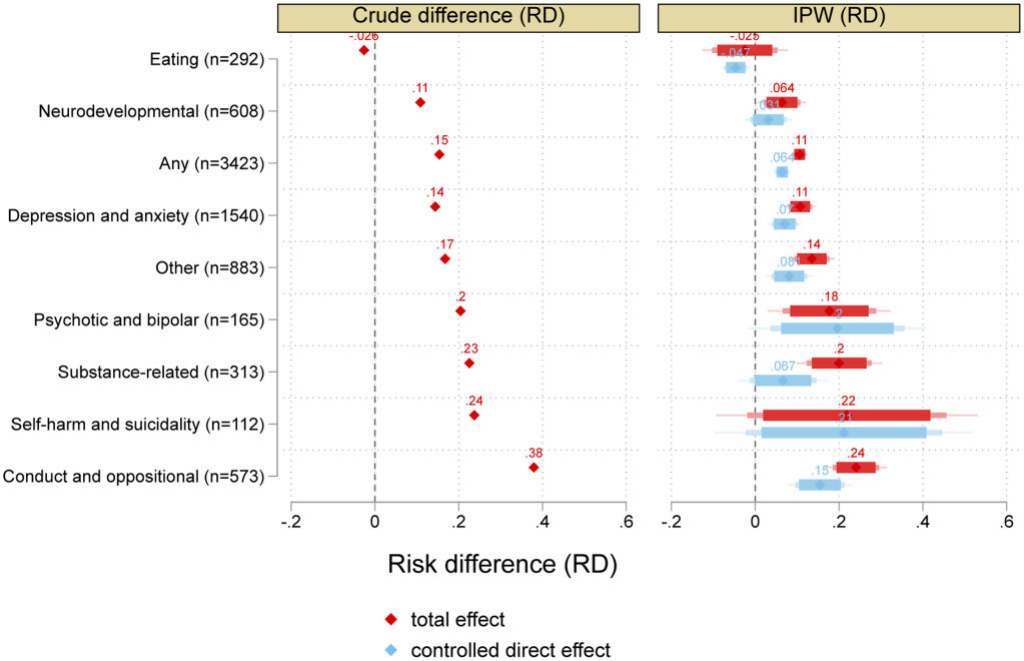
Crude differences, total effects and controlled direct effect (if all would complete upper secondary education) for each category. Separate models for each condition. Outcome: over-indebtedness at age 33. 99, 95 and 90% confidence intervals are shown. The risk ratios and proportion eliminated are shown in Supplementary table S4.

In figure 1, all specific disorders except eating disorders were linked to a higher risk of over-indebtedness. The crude association with over-indebtedness was greatest for conduct and opposite disorders (unadjusted RD = 0.38, RR = 4.53), self-harm (RD = 0.24, RR = 3.14) and substance use-related disorders (RD = 0.23, RR = 3.04). After balancing differences in childhood, parental and geographical characteristics, these associations were reduced but remained substantial in magnitude. Controlled direct effects suggested that the associations, particularly of substance use disorders, would be reduced to a significant extent had all completed upper secondary education.

## Discussion

We investigated the association between childhood psychiatric and neurodevelopmental disorders and adulthood over-indebtedness. People with records of adolescent diagnoses had a higher prevalence of over-indebtedness in their adulthood. This association remained substantial after balancing differences in the measured childhood, parental and geographical characteristics. The potential causal link between adolescent diagnoses and over-indebtedness operated, to a noteworthy extent, through the lower upper secondary education completion rate. Some 39% of the estimated effect would be reduced had all completed upper secondary education.

This is the first study to document an association between adolescent psychiatric and neurodevelopmental diagnoses and adulthood over-indebtedness. We found that the association was strong in magnitude and was observed among both sexes. An association between over-indebtedness and mental health is well established,^25^ yet studies have not considered a potential inverse pathway from earlier mental disorders to over-indebtedness. We estimated that adolescent diagnoses may cause a 2-fold risk of over-indebtedness but this finding relies on the critical assumption of no unmeasured confounding. However, there was large variation by diagnosis group.

While, to the best of our knowledge, no other studies have investigated debt-related outcomes, our finding is consistent with a larger body of literature showing that adolescent mental disorders predict economic difficulties later in life.^6^^,^^26^^,^^27^ For example, a Norwegian study^28^ suggested that mental health problems reduced adult income particularly at the lower end of the income distribution, thus causing significant financial difficulties.

We found that a critical pathway which linked the diagnoses in adolescence to over-indebtedness was the rate of upper secondary school completion. In line with the findings from this study, a substantial number of studies have shown that neurodevelopmental conditions and mental health problems increase the risk of dropping out of upper secondary school.^12–15^ Being without any formal education qualifications reduces the likelihood of stable employment, and thereby cause economic difficulties. Upper secondary schools may also provide skills and knowledge which are crucial for understanding the risks linked to various credit products and thus prevent over-indebtedness.

Several other mechanisms certainly play a role, but their role remains the topic for subsequent studies. For example, people may be targeted by the aggressive marketing of many short-term debt products and payment plans. Mental disorders and their stigma cause unemployment or work disability,^29^ which may lead to financial problems, and thus over-indebtedness. Treatment costs may cause additional economic strain. Nevertheless, examining the role of education, it may be that better education provides some protection against some of the harm caused by mental difficulties, in this case indicated by over-indebtedness.

Our findings signal that financial help may be useful to integrate with services for people with a psychiatric and neurodevelopmental diagnosis. Almost half of the people who were over-indebted had a history of psychiatric or neurodevelopmental diagnoses. This should be considered when designing debt help programmes and mental health support services. Moreover, programmes that increase the upper secondary school completion rate may be effective at reducing the adverse adulthood consequences of adolescent diagnoses. However, improving education alone may not be sufficient. Measures to promote neurodiverse society are needed to alleviate the increased risk of negative socioeconomic outcomes among people with neurodevelopmental conditions.

### Methodological considerations

The strengths of this study included register-based measures with no attrition and reporting bias, a low rate of missing data, a large population coverage and a rich set of control variables. Our study is thus not prone to non-response, misreporting and attrition issues typically present in studies on the adulthood consequences of childhood health.

However, our study did not cover the population born abroad, and people who emigrated in some parts of their life. Moreover, there may be some regional and socioeconomic factors affecting the diagnosing of psychiatric disorders in Finland. As a result, some measurement bias is possible, but we replicated the main analysis in each of the hospital districts. Fairly similar associations were observed in each hospital district (Supplementary figure S12).

Moreover, while we had access to rich set register data, we lacked information on many potential confounding factors. This is important limitation, and we suspect that some residual confounding bias certainly remains. For example, some early life risk behaviour may be an important confounding factor. We calculated E-values to assess the sensitivity for such bias.^30^ These indicated that an unmeasured confounder would need to have at least 3.4-fold (3- to 11-fold) effect on the RR scale, on both adolescent diagnosis and over-indebtedness in adulthood, conditional on measured covariates, to fully attenuate the total effect estimate.

## Conclusion

This full population study is the first to show that people affected by psychiatric and neurodevelopmental diagnoses in adolescence have a higher prevalence of over-indebtedness in adulthood. The association was observed for both men and women. Assuming no unmeasured confounding, this association may partly reflect a causal process. The potential causal link may operate to a large extent for a lower upper secondary completion rate of people with a psychiatric diagnosis. Policy measures, such as interventions to reduce non-completion rate of upper secondary education and measures to build a more neurodiverse society, are needed to prevent the over-indebtedness of people affected by adolescent psychiatric or neurodevelopmental disorders.

## Supplementary data

Supplementary data are available at *EURPUB* online.

## Funding

This work was supported by The Strategic Research Council at the Academy of Finland (grant number 312710), Academy of Finland (INVEST flagship project: grant number 320162). TL is supported by the Social Insurance Institution of Finland (grant number 29/26/2020).

*Conflicts of interest*: None declared.

## Supplementary Material

ckac126_Supplementary_Data

## Data Availability

Availability of the research data is subject to research permits from the Finnish Institute for Health and Welfare and respective register holders, mandated by Finnish data protection laws and the policies of the register holders. Stata 17 codes to replicate these findings are in the Open Science Framework (https://osf.io/49nmf/). We analyzed data from the full 1987 population birth cohort of Finland. People affected by an adolescent psychiatric or neurodevelopmental diagnosis had a 15 percentage points higher prevalence of over-indebtedness in adulthood. This association remained substantial after balancing differences in the measured pre-exposure childhood, parental and geographical characteristics. The potential causal link between adolescent diagnoses and over-indebtedness operated, to a noteworthy extent, through the lower upper secondary education completion rate.
