# Supplementary material for: Psychiatric and neurodevelopmental diagnoses in adolescence and adulthood over-indebtedness among Finns born in 1987
Source: Eur J Public Health. 2022 Oct 10;32(6):858–63. doi: 10.1093/eurpub/ckac126 (PMC9713378; doi:10.1093/eurpub/ckac126)
Supplement: ckac126_Supplementary_Data [file ckac126_supplementary_data.zip › ejph-2022-02-om-0090-File002.docx]

**Supplementary materials for *Psychiatric and neurodevelopmental disorders diagnoses in adolescence and adulthood over-indebtedness: the mediating role of upper secondary education among Finns born in 1987***

- Register sources of the variables used
- Sample selection
- Balance characteristics
- Separate models for each hospital district

Supplementary table 1. Description of the used variables. The Finnish birth cohort 1987.

| Variable | Variable type | Source | Definition |
| --- | --- | --- | --- |
| Outcome: Over–indebtedness | Dichotomous: yes, no | Register of debt payment entry records by Asiakastieto Ltd | Having at least 1 debt payment entry record in 1. December 2020. |
| Exposure: Psychiatric diagnosis at ages 13–17 (in 2000–4) | Dichotomous: yes, no | Care Register for Health Care by THL. https://thl.fi/en/web/thlfi–en/statistics/information–on–statistics/register–descriptions/care–register–for–health–care | Having at least 1 inpatient or outpatient hospitalization visit with codes F (excluding F0\|^F7\|^317\|^318\|^319) or X60–X84, Z72.8, Z91.5, Y87.0 in years 2000–2004 |
| Mediator: Upper secondary education qualifications | Dichotomous: yes, no | Education qualifications register by the Statistics of Finland |  |
| Birth date | Contionous (age), rang | Two sources:   - Social security number (VRK/DVV) - Medical birth register (THL) |  |
| Psychiatric diagnosis at ages 0–6 (in 1987–93) | Dichotomous: yes, no | Care Register for Health Care by THL. https://thl.fi/en/web/thlfi–en/statistics/information–on–statistics/register–descriptions/care–register–for–health–care |  |
| Psychiatric diagnosis at ages 7–12 (in 1994–9) | Dichotomous: yes, no | Care Register for Health Care by THL. https://thl.fi/en/web/thlfi–en/statistics/information–on–statistics/register–descriptions/care–register–for–health–care |  |
| Somatic diagnosis at ages 0–6 (in 1987–93) | Dichotomous: yes, no | Care Register for Health Care by THL. https://thl.fi/en/web/thlfi–en/statistics/information–on–statistics/register–descriptions/care–register–for–health–care |  |
| Somatic diagnosis at ages 7–12 (in 1994–9) | Dichotomous: yes, no | Care Register for Health Care by THL. https://thl.fi/en/web/thlfi–en/statistics/information–on–statistics/register–descriptions/care–register–for–health–care |  |
| Mother's age at birth | Contionous (age), range | Two sources:   - Social security number (VRK/DVV) - Medical birth register (THL) |  |
| Mother's somatic diagnosis at ages 0–12 of the cohort member (in 1987–99) | Dichotomous: yes, no | Care Register for Health Care by THL. https://thl.fi/en/web/thlfi–en/statistics/information–on–statistics/register–descriptions/care–register–for–health–care |  |
| Mother's psychiatric diagnosis at ages 0–12 of the cohort member (in 1987–99) | Dichotomous: yes, no | Care Register for Health Care by THL. https://thl.fi/en/web/thlfi–en/statistics/information–on–statistics/register–descriptions/care–register–for–health–care |  |
| Mother's hospitalisation visits at ages 0–12 of the cohort member (in 1987–99) | Dichotomous: yes, no | Care Register for Health Care by THL. https://thl.fi/en/web/thlfi–en/statistics/information–on–statistics/register–descriptions/care–register–for–health–care |  |
| Mother's work disability at ages 0–12 of the cohort member (in 1987–99) | Dichotomous: yes, no | Social Insurance Institution of Finland |  |
| Mother's unemployment duration at ages 0–12 of the cohort member (in 1987–99) | Months, range | Social Insurance Institution of Finland |  |
| Father's age at birth | Continuous; age, range | Two sources:   - Social security number (VRK/DVV) - Medical birth register (THL) |  |
| Father's somatic diagnosis at ages 0–12 of the cohort member (in 1987–99) | Dichotomous: yes, no | Care Register for Health Care by THL. https://thl.fi/en/web/thlfi–en/statistics/information–on–statistics/register–descriptions/care–register–for–health–care |  |
| Father's psychiatric diagnosis at ages 0–12 of the cohort member (in 1987–99) | Dichotomous: yes, no | Care Register for Health Care by THL. https://thl.fi/en/web/thlfi–en/statistics/information–on–statistics/register–descriptions/care–register–for–health–care |  |
| Father's hospitalisation visits at ages 0–12 of the cohort member (in 1987–99) | Dichotomous: yes, no | Care Register for Health Care by THL. https://thl.fi/en/web/thlfi–en/statistics/information–on–statistics/register–descriptions/care–register–for–health–care |  |
| Father's work disability at ages 0–12 of the cohort member (in 1987–99) | Dichotomous: yes, no | Social Insurance Institution of Finland |  |
| Father's unemployment duration at ages 0–12 of the cohort member (in 1987–99) |  | Social Insurance Institution of Finland |  |
| Death of a parent at ages 0–12 of the cohort member (in 1987–99) |  | Statistics of Finland |  |
| Parents social assistance use at ages 0–12 of the cohort member (in 1987–99) |  | - Register of means tested benefits (Toimentulotukirekisteri by THL) |  |
| Placement outside home before the age of 13 |  | - Register of Child Welfare by THL: https://thl.fi/en/web/thlfi-en/statistics-and-data/data-and-services/register-descriptions/child-welfare |  |
| Birth sex |  | Two sources:   - Social security number (VRK/DVV) - Last known gender |  |
| Birth weight |  | - Medical birth register THL |  |
| No data on father's age |  | Derived from other variables |  |
| Municipatal level unemployment in 1999 |  | Statistics of Finland. |  |

Supplementary table 2. Description of the sample selection. The Finnish birth cohort 1987.

|  | ALL | No psychiatric records in 2000–4 | Psychiatric records in 2000–4 |
| --- | --- | --- | --- |
| **Finnish Birth Cohort of 1987** | 59476 |  |  |
| – deceased in 1987–2004 | -347 |  |  |
| – immigrated in 1987–2004 | -837 |  |  |
| – Excluded due to F0\|^F7\|^317\|^318\|^319 | -190 |  |  |
| **Observed in 2000–4** | 58102 | 54156 | 3946 |
| – deceased in 2005–2020 | -598 | -463 | -135 |
| – immigrated in 2005–2020 | -1940 | -1855 | -85 |
| – records of conservartorship | -289 | -163 | -126 |
| – missing values in confounders | -1532 | -1355 | -177 |
| **Analysed sample** | 53743 | 50320 | 3423 |

Supplementary table 3. Description of the sample by adolescence psychiatric diagnosis before and after weighting. The Finnish birth cohort 1987. (päivitä)

|  | Unweighted | | | IPTW weighted | | |
| --- | --- | --- | --- | --- | --- | --- |
|  | No psychiatric diagnosis at aged 12–17 | Psychiatric diagnosis at aged 12–17 | Standardised difference | No psychiatric diagnosis at aged 12–17 | Psychiatric diagnosis at aged 12–17 | Standardised difference |
| Number of people | 50347 | 3396 | – | – | – | – |
| Mediator (not included in the IPTW weighting) |  |  |  |  |  |  |
| Upper secondary education qualifications |  |  |  |  |  |  |
| Yes | 0.92 | 0.74 | –0.49 | 0.91 | 0.78 | –0.39 |
| Control variables (included in the IPTW weighting) |  |  |  |  |  |  |
| Age when over–indebtedness was measured |  |  |  |  |  |  |
|  | 32.51 | 32.51 | 0.01 | 32.51 | 32.50 | –0.02 |
| Psychiatric diagnosis at ages 0–6 (in 1987–93) |  |  |  |  |  |  |
|  | 0.01 | 0.02 | 0.14 | 0.01 | 0.01 | 0.04 |
| Psychiatric diagnosis at ages 7–12 (in 1994–9) |  |  |  |  |  |  |
|  | 0.01 | 0.04 | 0.23 | 0.01 | 0.01 | 0.01 |
| Somatic diagnosis at ages 0–6 (in 1987–93) |  |  |  |  |  |  |
|  | 0.37 | 0.44 | 0.13 | 0.38 | 0.37 | –0.01 |
| Somatic diagnosis at ages 7–12 (in 1994–9) |  |  |  |  |  |  |
|  | 0.34 | 0.50 | 0.32 | 0.35 | 0.36 | 0.02 |
| Mother's age at birth |  |  |  |  |  |  |
|  | 28.96 | 28.30 | –0.12 | 28.92 | 28.92 | 0.00 |
| Mother's somatic diagnosis at ages 0–12 of the cohort member (in 1987–99) |  |  |  |  |  |  |
|  | 0.69 | 0.76 | 0.16 | 0.70 | 0.69 | –0.00 |
| Mother's psychiatric diagnosis at ages 0–12 of the cohort member (in 1987–99) |  |  |  |  |  |  |
|  | 0.04 | 0.09 | 0.20 | 0.04 | 0.04 | 0.02 |
| Mother's hospitalisation visits at ages 0–12 of the cohort member (in 1987–99) |  |  |  |  |  |  |
|  | 3.50 | 5.29 | 0.19 | 3.62 | 3.72 | 0.01 |
| Mother's work disability at ages 0–12 of the cohort member (in 1987–99) |  |  |  |  |  |  |
|  | 0.01 | 0.03 | 0.09 | 0.01 | 0.01 | 0.01 |
| Mother's unemployment duration at ages 0–12 of the cohort member (in 1987–99) |  |  |  |  |  |  |
|  | 0.55 | 0.86 | 0.20 | 0.57 | 0.58 | 0.01 |
| Father's age at birth |  |  |  |  |  |  |
|  | 31.29 | 30.93 | –0.06 | 31.27 | 31.38 | 0.02 |
| Father's somatic diagnosis at ages 0–12 of the cohort member (in 1987–99) |  |  |  |  |  |  |
|  | 0.57 | 0.61 | 0.09 | 0.57 | 0.57 | 0.00 |
| Father's psychiatric diagnosis at ages 0–12 of the cohort member (in 1987–99) |  |  |  |  |  |  |
|  | 0.05 | 0.10 | 0.18 | 0.05 | 0.06 | 0.01 |
| Father's hospitalisation visits at ages 0–12 of the cohort member (in 1987–99) |  |  |  |  |  |  |
|  | 2.89 | 3.80 | 0.11 | 2.94 | 2.98 | 0.00 |
| Father's work disability at ages 0–12 of the cohort member (in 1987–99) |  |  |  |  |  |  |
|  | 0.02 | 0.04 | 0.10 | 0.02 | 0.03 | 0.01 |
| Father's unemployment duration at ages 0–12 of the cohort member (in 1987–99) |  |  |  |  |  |  |
|  | 0.49 | 0.91 | 0.23 | 0.52 | 0.55 | 0.02 |
| Death of a parent at ages 0–12 of the cohort member (in 1987–99) |  |  |  |  |  |  |
|  | 0.02 | 0.05 | 0.12 | 0.03 | 0.03 | 0.01 |
| Parents social assistance use at ages 0–12 of the cohort member (in 1987–99) |  |  |  |  |  |  |
|  | 8.50 | 19.78 | 0.37 | 9.27 | 9.97 | 0.03 |
| Birth sex |  |  |  |  |  |  |
| Men | 0.52 | 0.41 | –0.23 | 0.51 | 0.50 | –0.03 |
| No data on father's age |  |  |  |  |  |  |
|  | 0.01 | 0.02 | 0.09 | 0.01 | 0.01 | 0.02 |
| Placement outside home before the age of 13 |  |  |  |  |  |  |
|  | 0.01 | 0.04 | 0.21 | 0.01 | 0.01 | 0.01 |
| Local unemployment rate in 1999 |  |  |  |  |  |  |
|  | 14.55 | 13.79 | –0.16 | 14.51 | 14.65 | 0.03 |
| Birth weight |  |  |  |  |  |  |
|  | 3577.02 | 3515.02 | –0.11 | 3572.77 | 3562.28 | –0.02 |
|  |  |  |  |  |  |  |

Supplementary table 4. Estimates for specific psychiatric and neurodevelopmental disorders.

|  | any_f10f99 | neurodev | conduct_and_oppos | self_harm | substance | depr_and_anxiety | other_fdiag | eating | psycho_and_bipo |
| --- | --- | --- | --- | --- | --- | --- | --- | --- | --- |
|  | b/ci95 | b/ci95 | b/ci95 | b/ci95 | b/ci95 | b/ci95 | b/ci95 | b/ci95 | b/ci95 |
| crude_difference | 0.15 | 0.11 | 0.38 | 0.24 | 0.23 | 0.14 | 0.17 | -0.03 | 0.20 |
|  | 0.14,0.17 | 0.08,0.14 | 0.34,0.42 | 0.14,0.33 | 0.17,0.28 | 0.12,0.17 | 0.14,0.20 | -0.06,0.01 | 0.13,0.27 |
| RR_crude | 2.51 | 1.98 | 4.53 | 3.14 | 3.04 | 2.34 | 2.54 | 0.77 | 2.84 |
|  | 2.36,2.67 | 1.69,2.28 | 4.14,4.92 | 2.28,4.00 | 2.58,3.51 | 2.13,2.55 | 2.26,2.82 | 0.48,1.05 | 2.21,3.47 |
| treated | 0.21 | 0.17 | 0.35 | 0.33 | 0.31 | 0.22 | 0.24 | 0.09 | 0.29 |
|  | 0.20,0.22 | 0.13,0.22 | 0.29,0.40 | 0.09,0.57 | 0.23,0.39 | 0.19,0.24 | 0.20,0.29 | 0.01,0.16 | 0.18,0.40 |
| comparison | 0.10 | 0.11 | 0.11 | 0.11 | 0.11 | 0.11 | 0.11 | 0.11 | 0.11 |
|  | 0.10,0.11 | 0.11,0.11 | 0.11,0.11 | 0.11,0.12 | 0.11,0.11 | 0.11,0.11 | 0.11,0.11 | 0.11,0.11 | 0.11,0.11 |
| RD_ | 0.11 | 0.06 | 0.24 | 0.22 | 0.20 | 0.11 | 0.14 | -0.03 | 0.18 |
|  | 0.09,0.12 | 0.02,0.11 | 0.19,0.30 | -0.02,0.46 | 0.12,0.28 | 0.08,0.14 | 0.09,0.18 | -0.10,0.05 | 0.07,0.29 |
| risk_ratio | 2.01 | 1.57 | 3.22 | 2.97 | 2.81 | 1.99 | 2.23 | 0.78 | 2.58 |
|  | 1.86,2.16 | 1.18,1.97 | 2.70,3.73 | 0.81,5.13 | 2.11,3.51 | 1.73,2.25 | 1.85,2.62 | 0.07,1.48 | 1.58,3.59 |
| controlled_treated | 0.14 | 0.11 | 0.24 | 0.29 | 0.15 | 0.15 | 0.16 | 0.04 | 0.28 |
|  | 0.13,0.16 | 0.07,0.16 | 0.18,0.29 | 0.06,0.53 | 0.07,0.23 | 0.12,0.18 | 0.12,0.20 | 0.01,0.06 | 0.12,0.44 |
| controlled_comparising | 0.08 | 0.08 | 0.08 | 0.08 | 0.08 | 0.08 | 0.08 | 0.08 | 0.08 |
|  | 0.08,0.08 | 0.08,0.09 | 0.08,0.08 | 0.08,0.09 | 0.08,0.08 | 0.08,0.08 | 0.08,0.08 | 0.08,0.09 | 0.08,0.09 |
| CDE_rd | 0.06 | 0.03 | 0.15 | 0.21 | 0.07 | 0.07 | 0.08 | -0.05 | 0.20 |
|  | 0.05,0.08 | -0.01,0.07 | 0.10,0.21 | -0.02,0.45 | -0.01,0.15 | 0.04,0.10 | 0.04,0.12 | -0.07,-0.02 | 0.04,0.36 |
| CDE_rr | 1.82 | 1.38 | 2.90 | 3.57 | 1.81 | 1.87 | 2.00 | 0.43 | 3.38 |
|  | 1.61,2.02 | 0.85,1.91 | 2.17,3.64 | 0.69,6.45 | 0.83,2.79 | 1.49,2.25 | 1.47,2.52 | 0.11,0.76 | 1.43,5.33 |
| PE | 0.39 | 0.51 | 0.36 | 0.03 | 0.67 | 0.35 | 0.40 | -0.86 | -0.11 |
|  | 0.28,0.51 | -3.29,4.32 | 0.16,0.56 | -2.82,2.89 | 0.32,1.01 | 0.12,0.58 | 0.17,0.62 | -71.35,69.63 | -0.93,0.71 |
| N | 53743 | 53743 | 53743 | 53743 | 53743 | 53743 | 53743 | 53743 | 53743 |


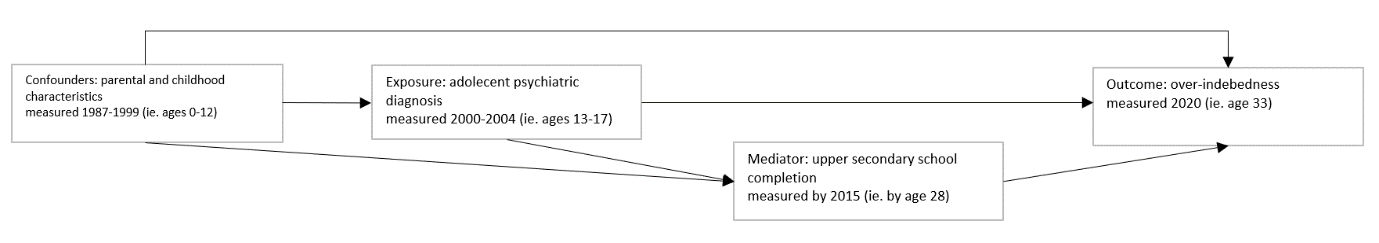


Supplementary Figure 1. Directed acyclic graph for the measured variables and assumed causal paths.


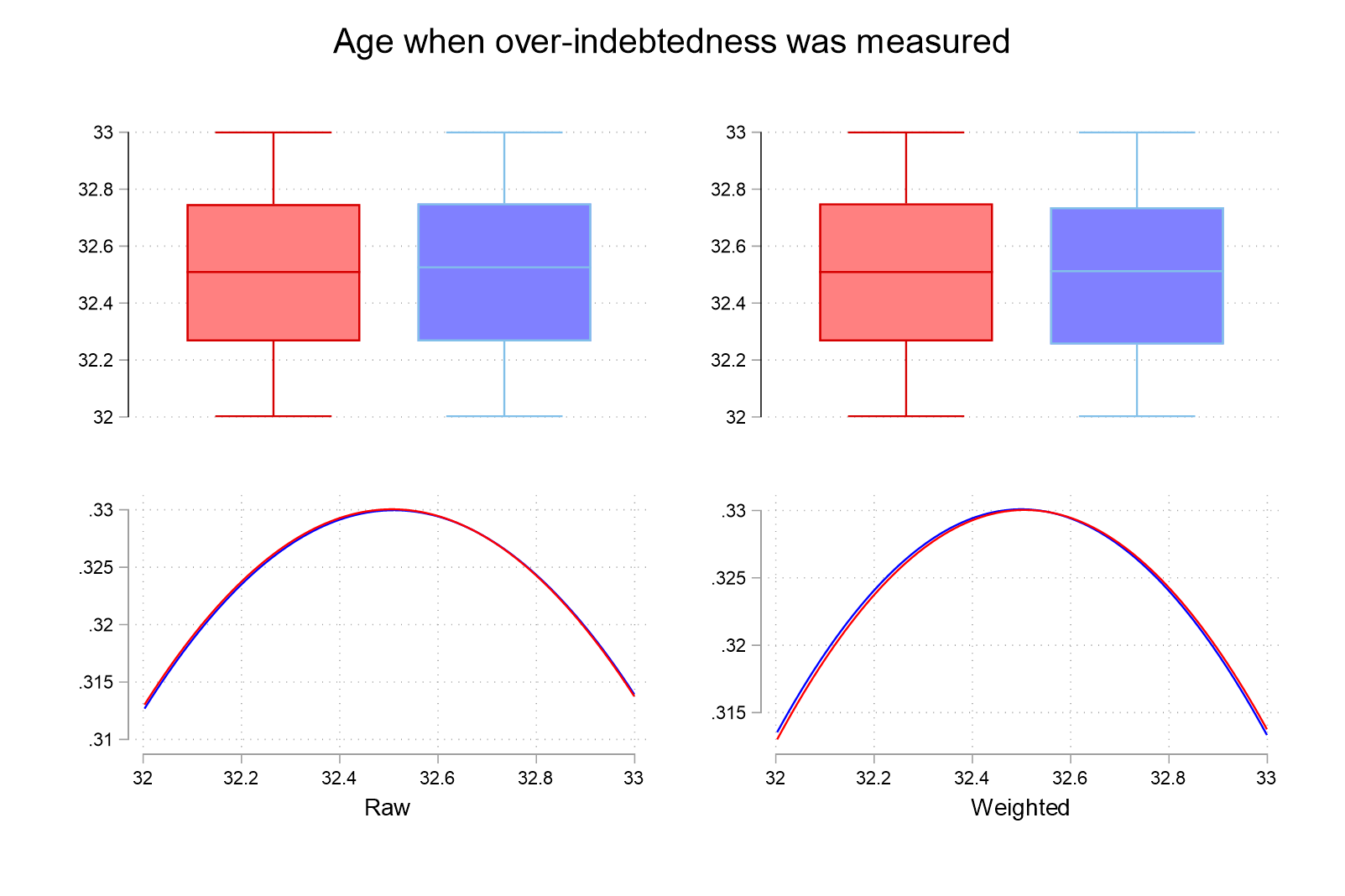


Supplementary figure 2. Balance in Age when over-indebtedness was measured before and after weighting. Blue is treated and red is untreated.


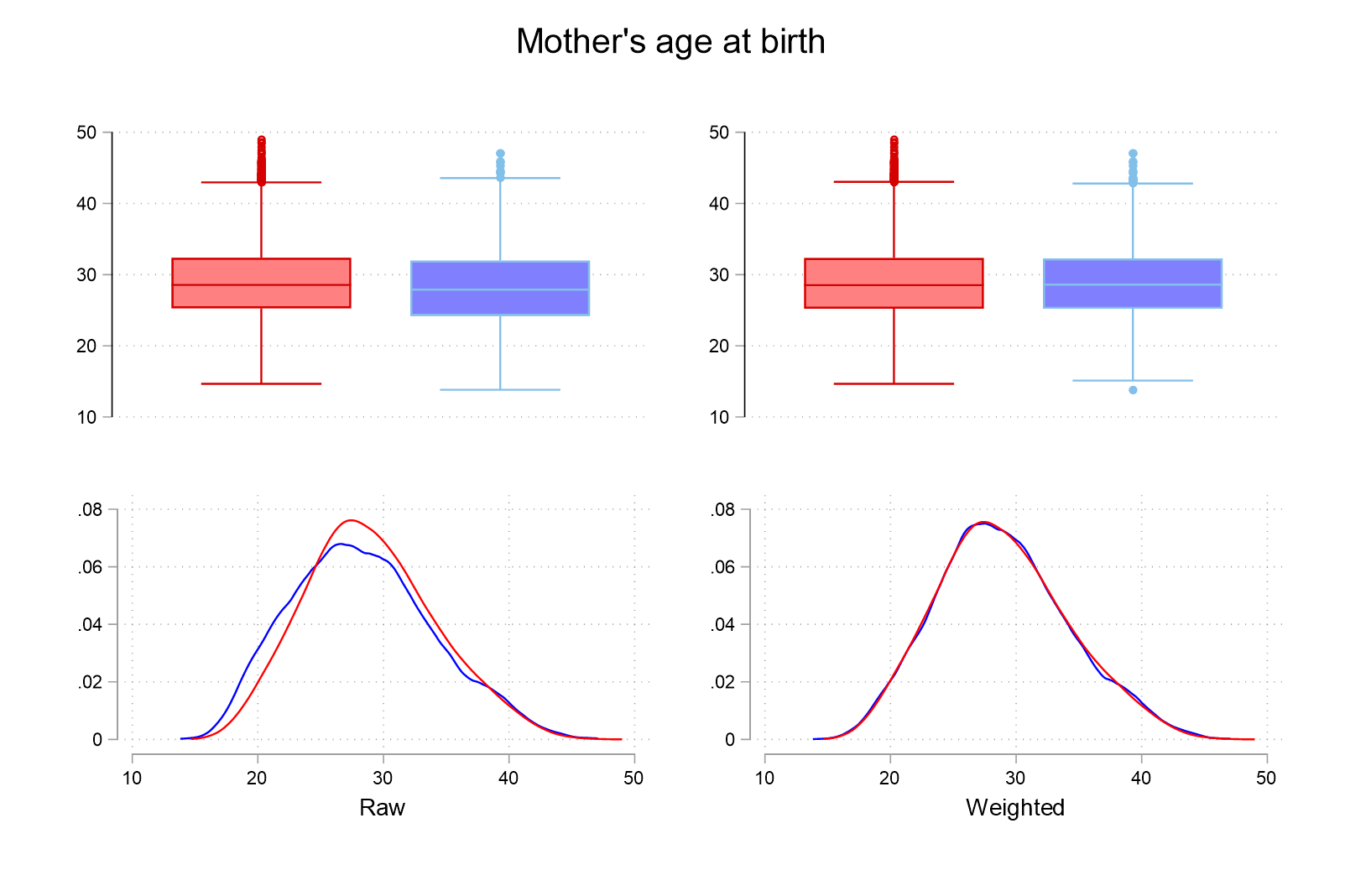


Supplementary figure 3. Balance in Mother's age at birth before and after weighting. Blue is treated and red is untreated.


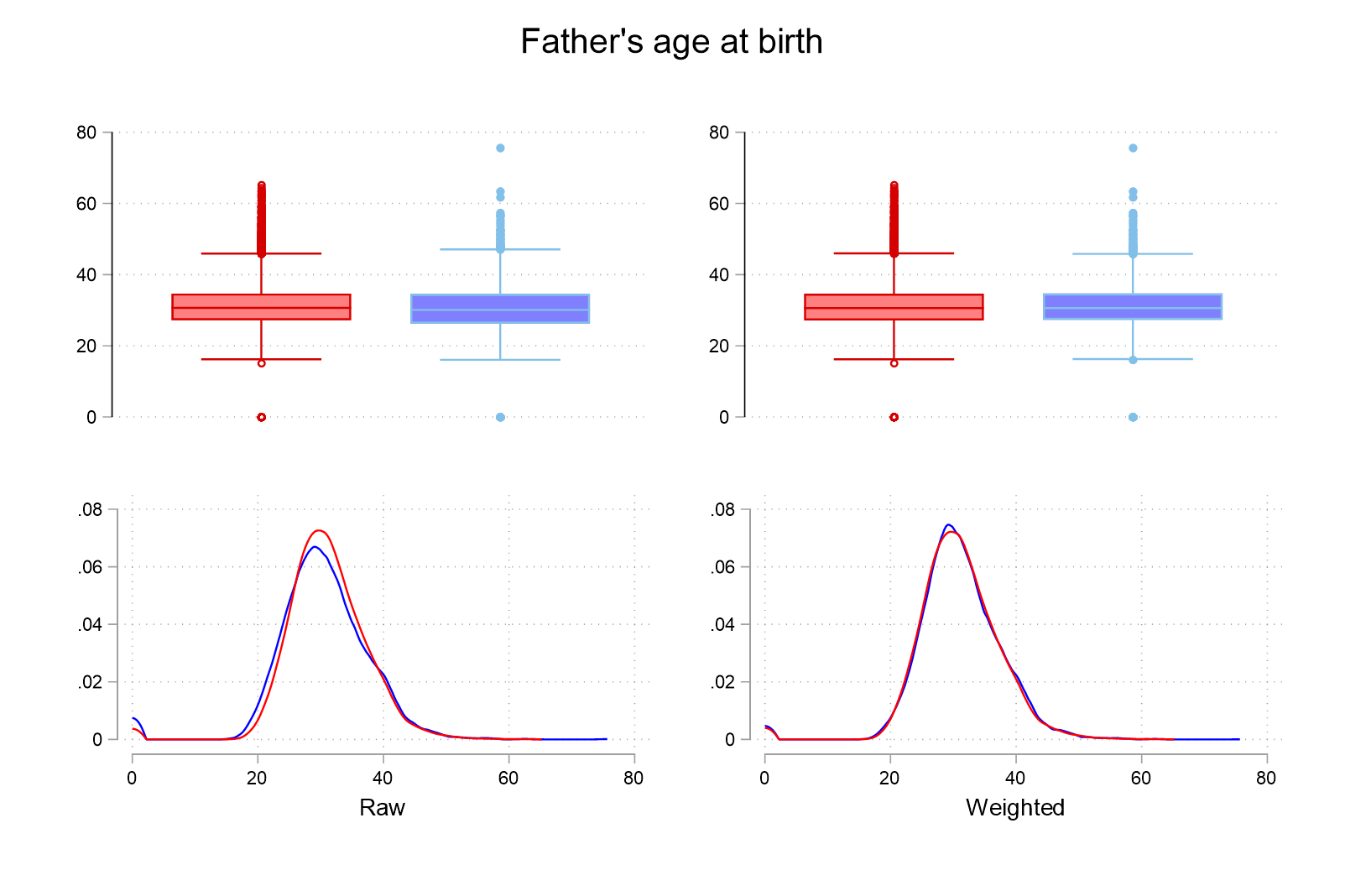


Supplementary figure 4. Balance in Father's age at birth before and after weighting. Blue is treated and red is untreated.


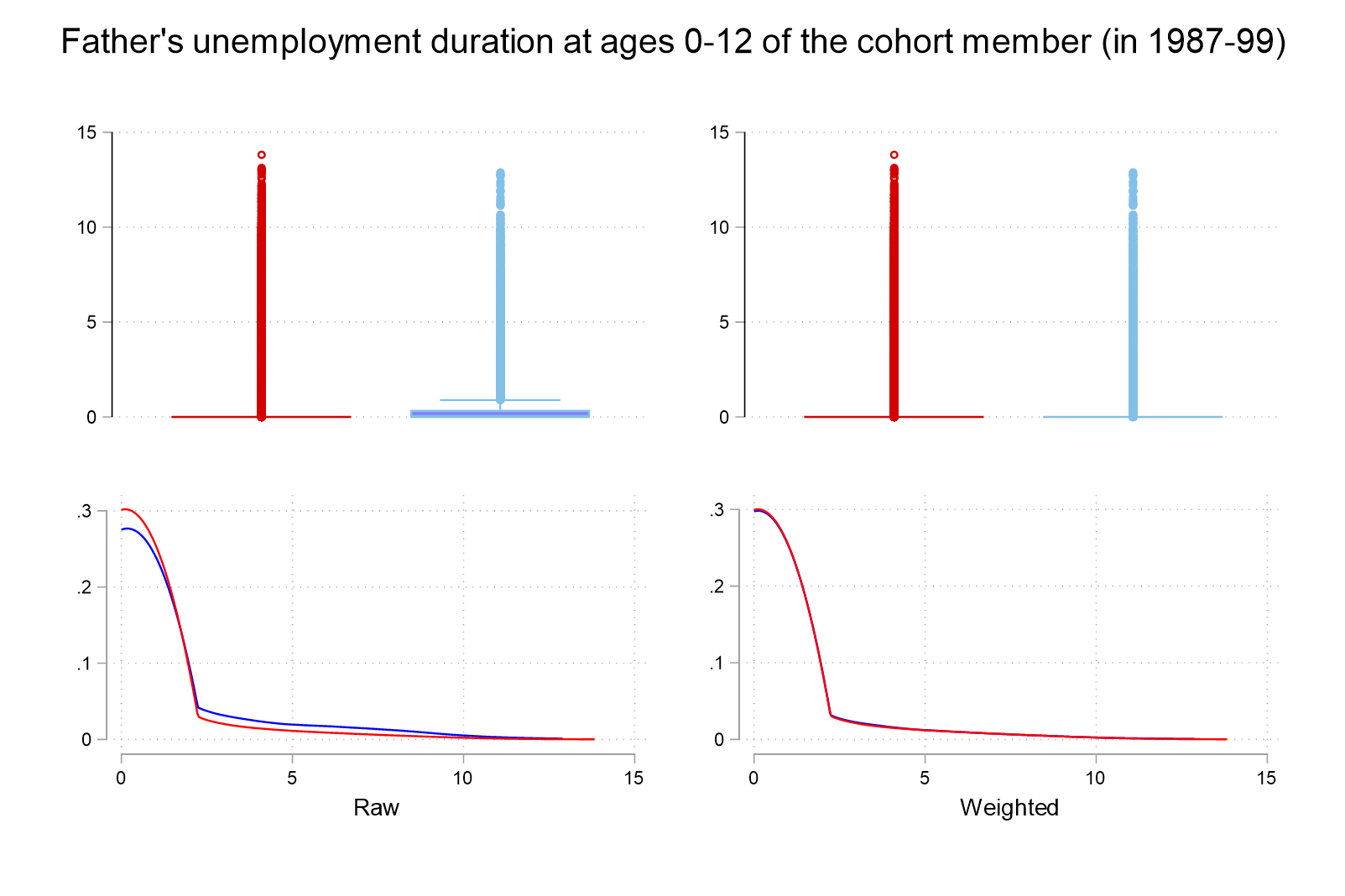


Supplementary figure 5. Balance in Father's unemployment duration at ages 0-12 of the cohort member (in 1987-99) before and after weighting. Blue is treated and red is untreated.


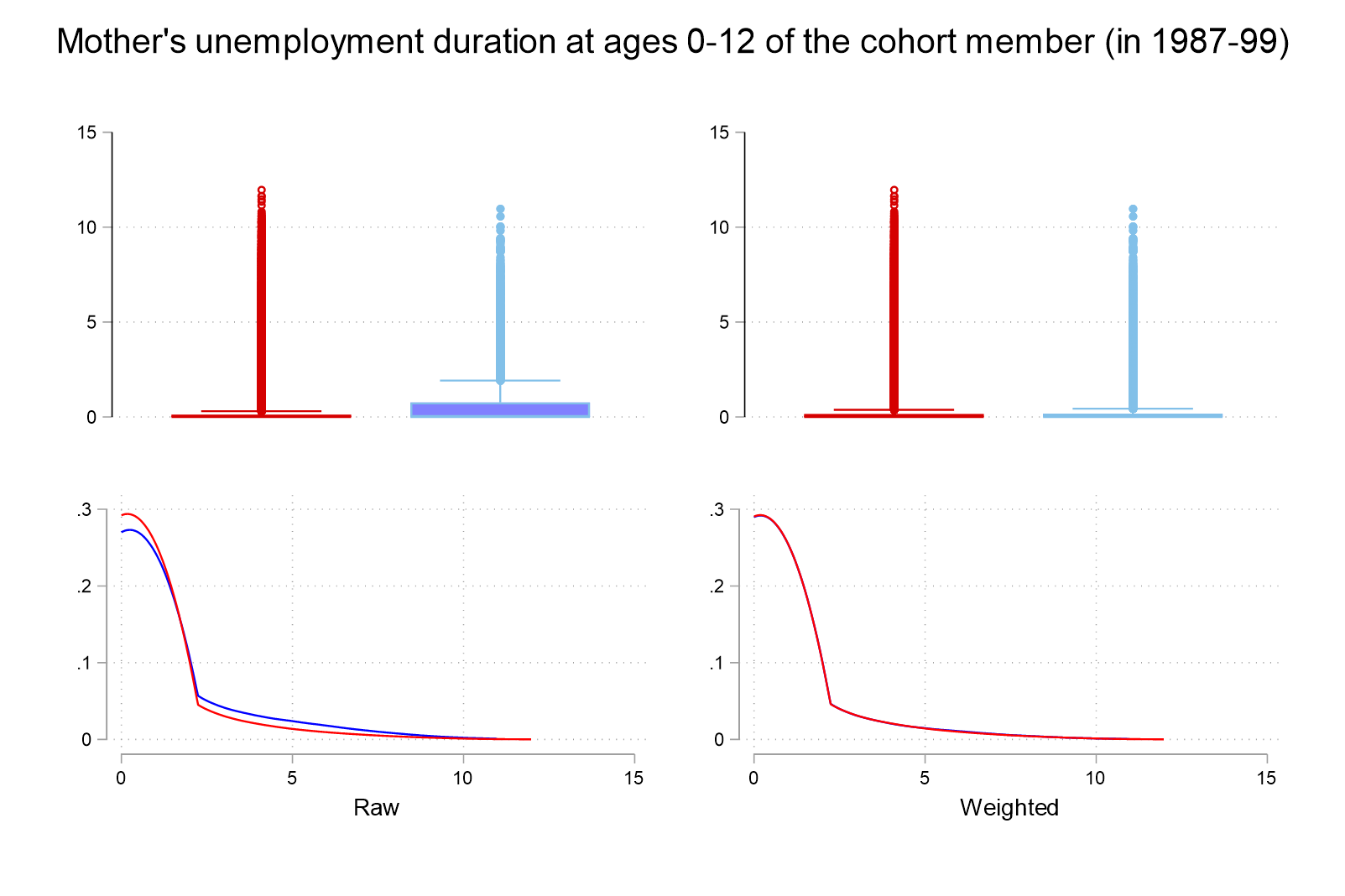


Supplementary figure 6. Balance in Mother's unemployment duration at ages 0-12 of the cohort member (in 1987-99) before and after weighting. Blue is treated and red is untreated.


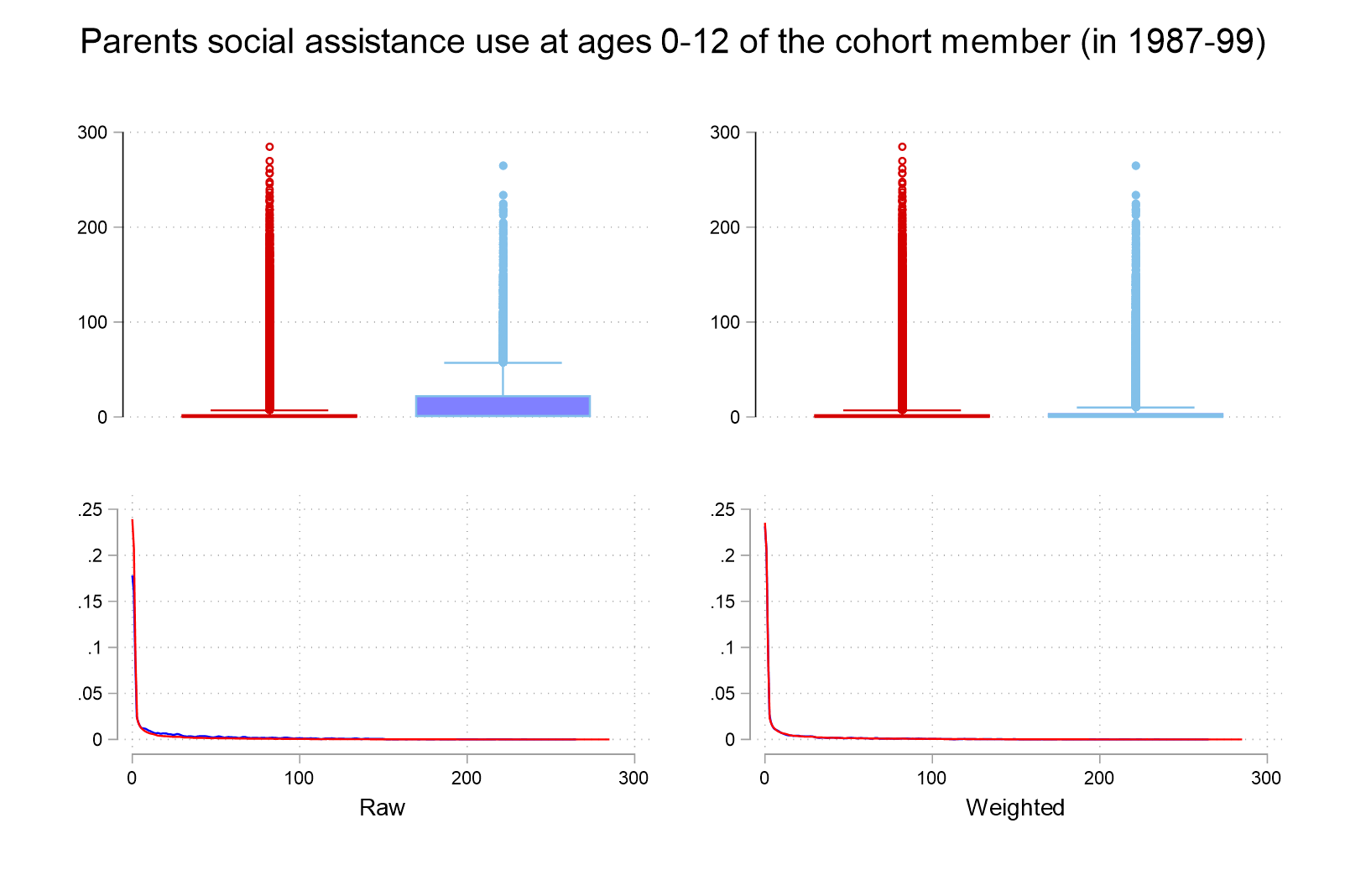


Supplementary figure 7. Balance in Parents social assistance use at ages 0-12 of the cohort member (in 1987-99) before and after weighting. Blue is treated and red is untreated.


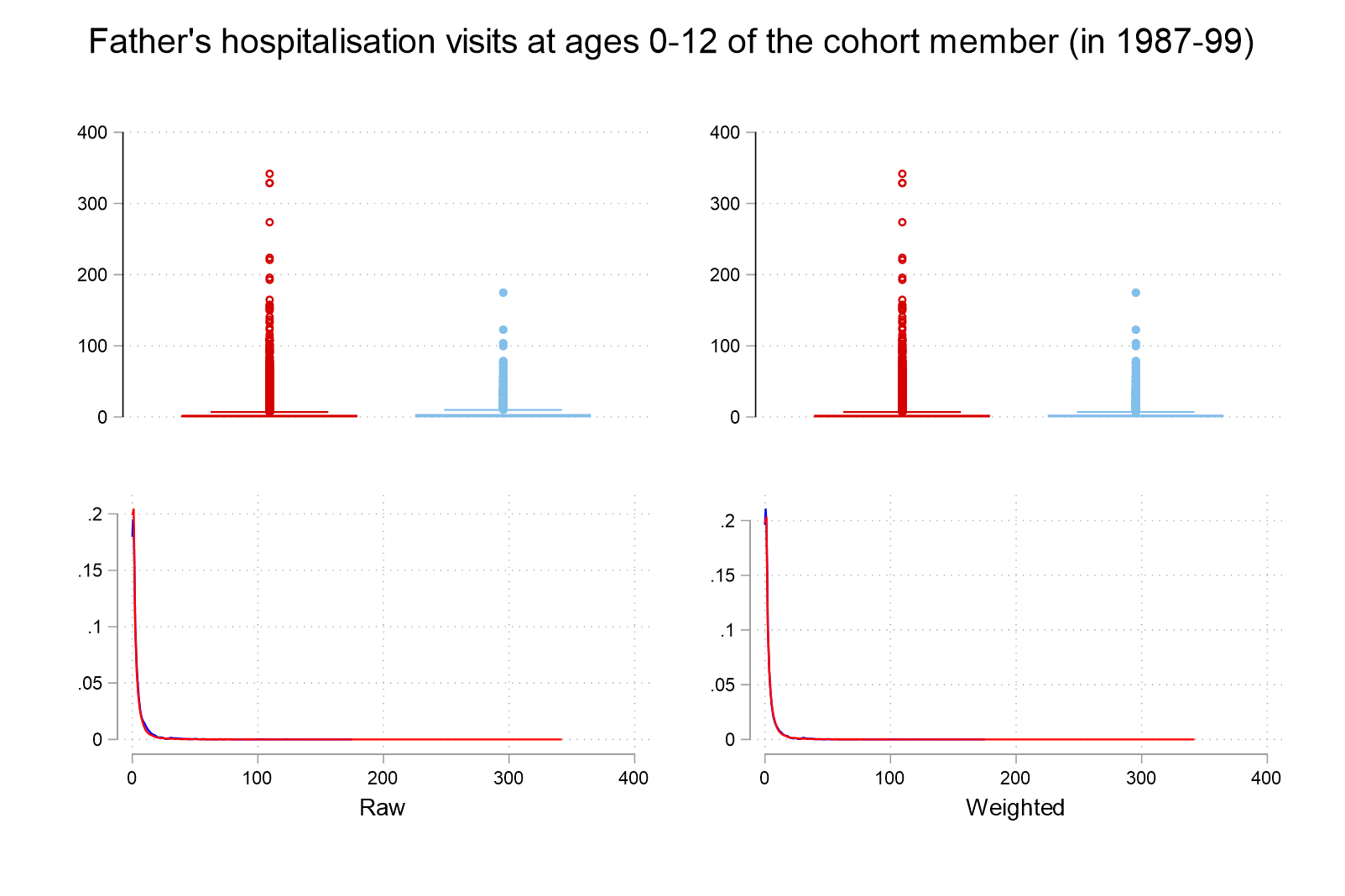


Supplementary figure 8. Balance in Father's hospitalisation visits at ages 0-12 of the cohort member (in 1987-99) before and after weighting. Blue is treated and red is untreated.


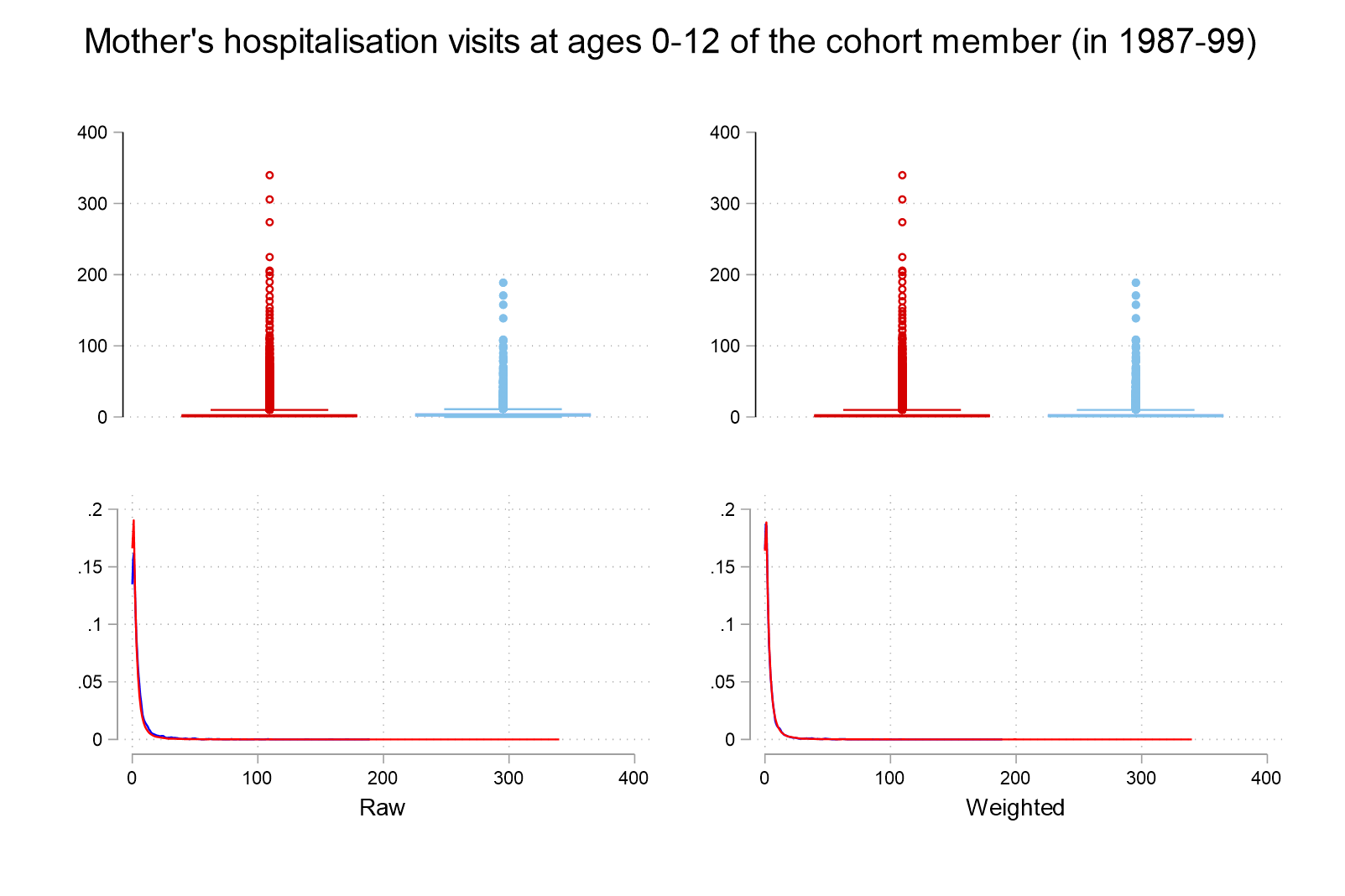


Supplementary figure 9. Balance in Mother's hospitalisation visits at ages 0-12 of the cohort member (in 1987-99) before and after weighting. Blue is treated and red is untreated.


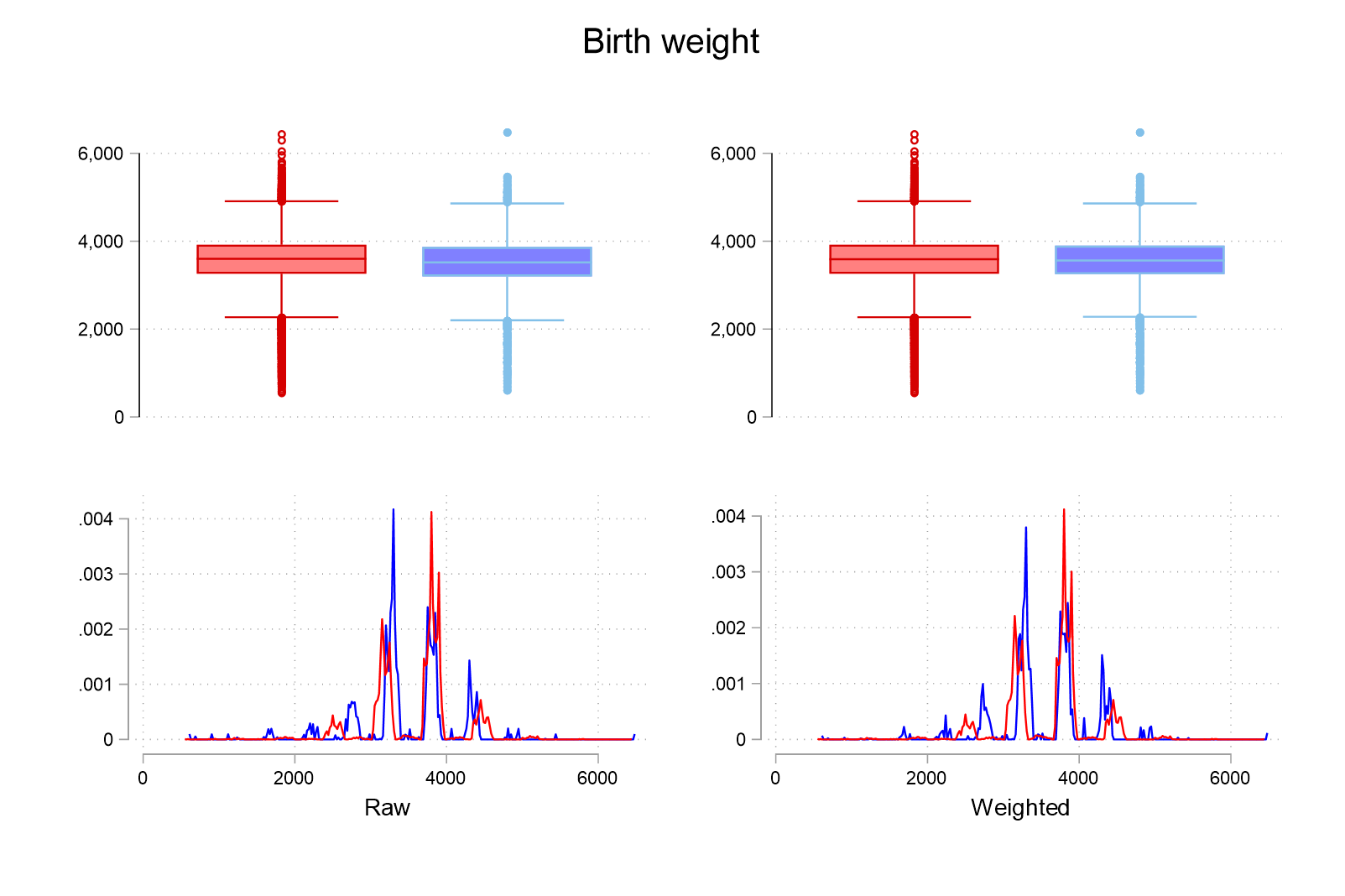


Supplementary figure 10. Balance in Birth weight before and after weighting. Blue is treated and red is untreated.


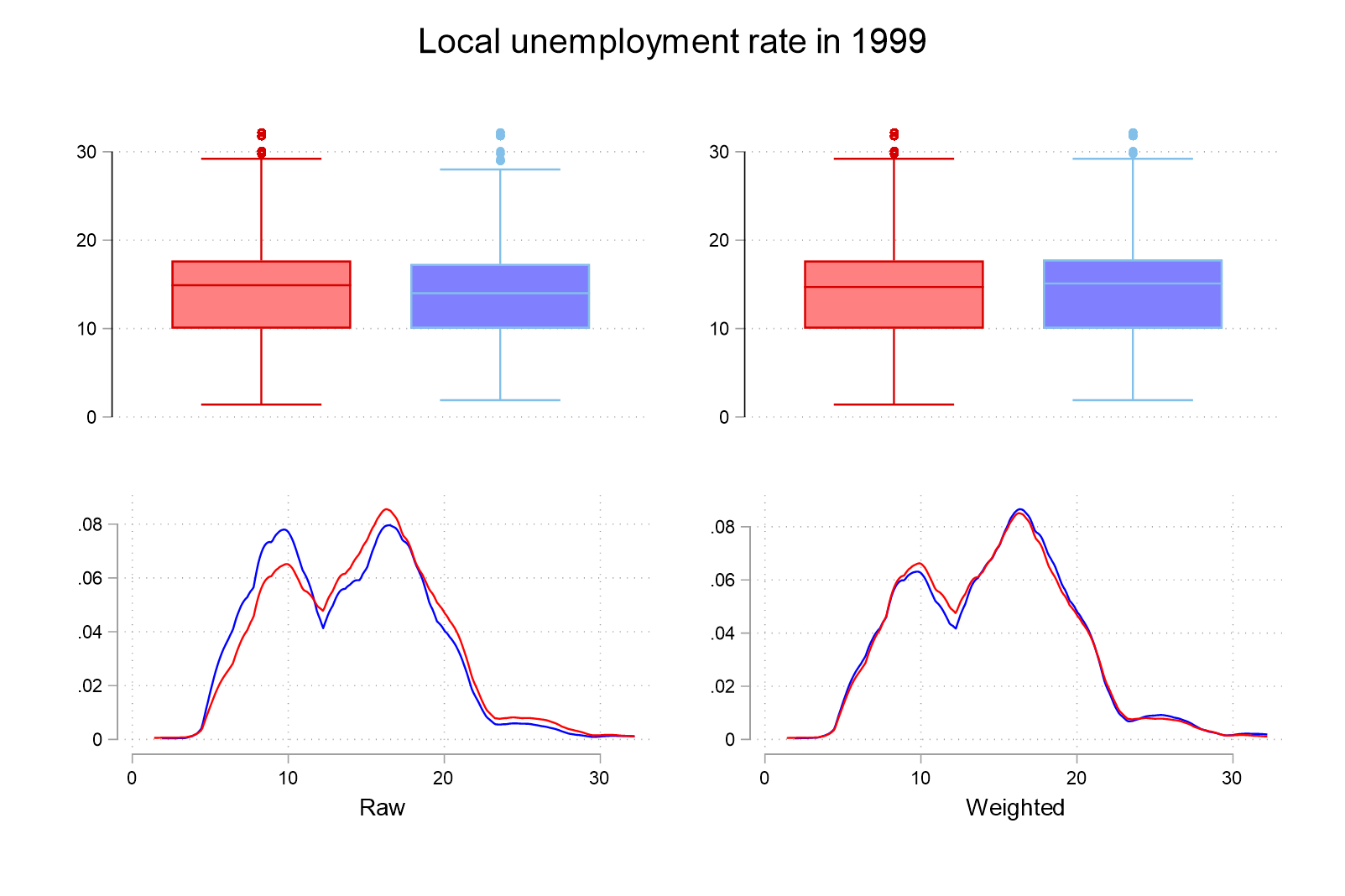


Supplementary figure 11. Balance in Local unemployment rate in 1999 before and after weighting. Blue is treat


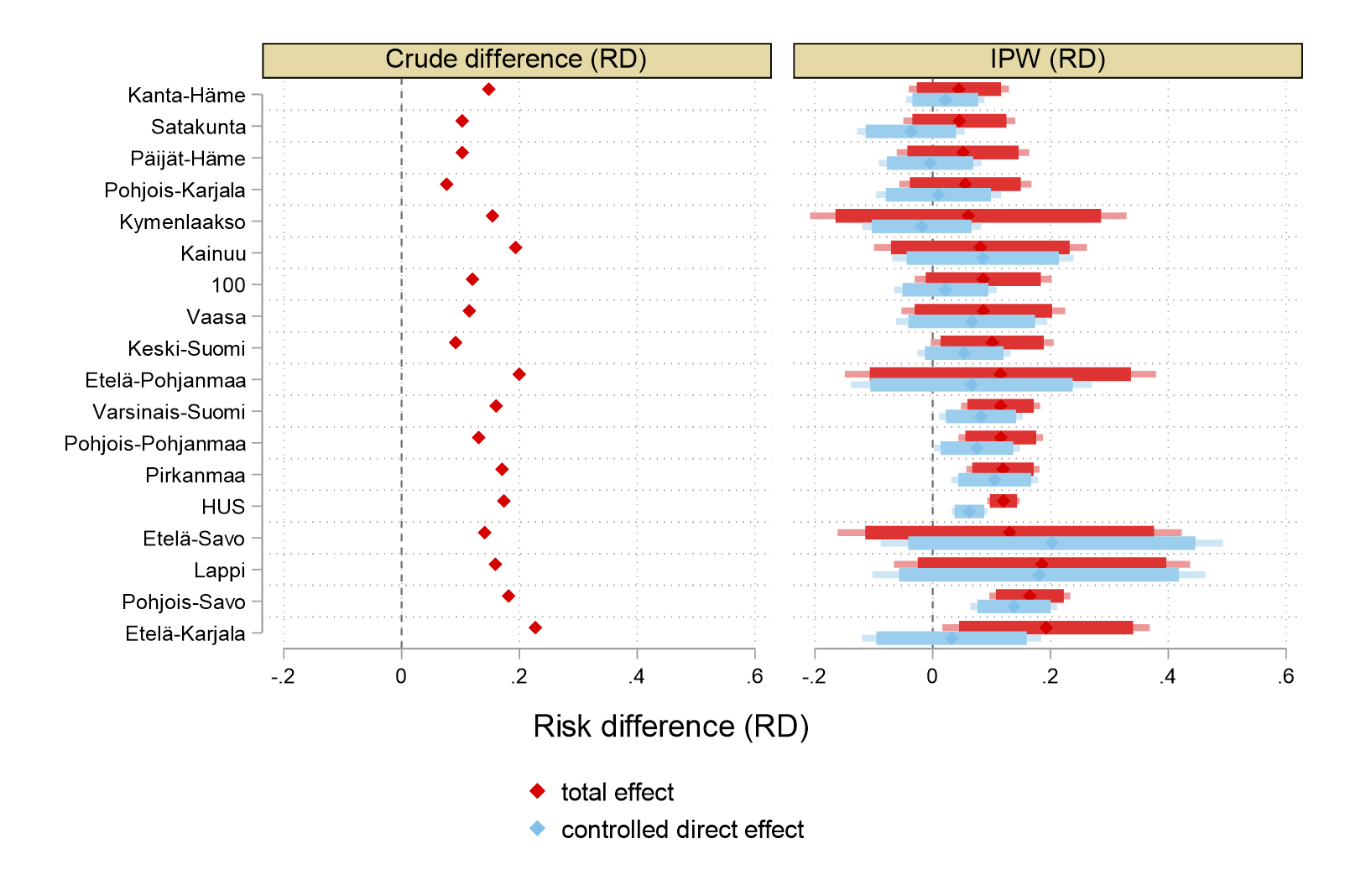


Supplementary figure 12. Crude differences, total effects and controlled direct effects of adolescence psychiatric diagnosis on adulthood over–indebtedness by hospital districts. Separate models. 100=all other, smaller, hospital districts. Standard errors calculated using bootstrapping.
